# Cross-Linking/Mass Spectrometry Uncovers Details of Insulin-Like Growth Factor Interaction With Insect Insulin Binding Protein Imp-L2

**DOI:** 10.3389/fendo.2019.00695

**Published:** 2019-10-09

**Authors:** Petr Pompach, Cristina M. Viola, Jelena Radosavljević, Jingjing Lin, Jiří Jiráček, Andrzej M. Brzozowski, Irena Selicharová

**Affiliations:** ^1^Institute of Microbiology, Czech Academy of Sciences, Prague, Czechia; ^2^York Structural Biology Laboratory, Department of Chemistry, The University of York, York, United Kingdom; ^3^Institute of Organic Chemistry and Biochemistry, Czech Academy of Sciences, Prague, Czechia

**Keywords:** IGF-1, Imp-L2, cross-linking, diazirine ring, mass spectrometry

## Abstract

Structural details of changes accompanying interaction between insulin-related hormones and their binding partners are often enigmatic. Here, cross-linking/mass spectrometry could complement structural techniques and reveal details of these protein-protein interfaces. We used such approach to clarify missing structural description of the interface in human insulin-like growth factor (IGF-1): *Drosophila melanogaster* imaginal morphogenesis protein-late 2 protein (Imp-L2) complex which we studied previously by X-ray crystallography. We crosslinked these proteins by heterobifunctional cross-linker sulfosuccinimidyl 4,4′-azidopentanoate (Sulfo-SDA) for the subsequent mass spectrometry (MS) analysis. The MS analysis revealed IGF-1:Imp-L2 interactions which were not resolved in the crystal structure of this assembly, and they converged with X-ray results, indicating the importance of the IGF-1 N-terminus interaction with the *C*-terminal (185–242) part of the Imp-L2 for stability of this complex. Here, we also showed the advantage and reliability of MS approach in solving details of protein-protein interactions that are too flexible for solid state structural methods.

## Introduction

The insulin/insulin-like growth factor signaling axis is an evolutionary ancient and highly conserved hormonal system involved in the regulation of metabolism, growth and lifespan in animals. In humans this axis contains insulin and two insulin-like growth factors (IGF-1 and IGF-2) eliciting their function through activation of tyrosine kinase-type receptors (1). Secretion of insulin from pancreatic β-cells is regulated in response to blood glucose levels. Subsequently, the active monomeric form of this hormone circulates freely in the blood and is readily cleared away in minutes. In contrast, IGFs are secreted by many tissues in endocrine or paracrine mode and their bioavailability is controlled by several IGF binding proteins (IGFBP 1–6) (2) and the non-signaling IGF-2/mannose-6-phosphate receptor (3). There are also IGFBP-related proteins (IGFBP-rP) that carry the *N*-terminal domain of IGFBPs hence are classified as part of the IGFBP superfamily. However, IGFBP-rPs have low or no affinity for IGFs/insulin, and their functions are not fully elucidated being, most probably, IGF/insulin independent (4).

**Figure 1:**
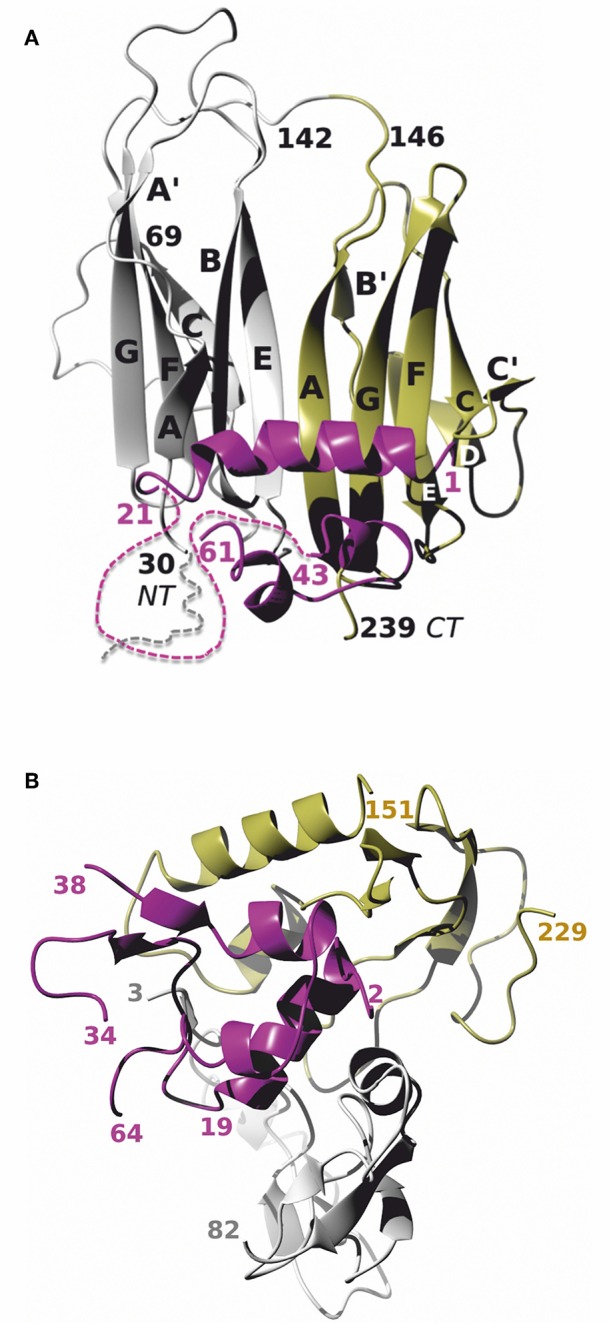
**(A)** The crystal structure of Imp-L2:IGF-1 complex (PDB ID 6FF3). Imp-L2 Ig-NT and Ig-CT domains are in gray and green, respectively, and IGF-1 with its numbering is in magenta; A–G refer to the β-strand nomenclature as in Roed et al. (7). Some parts of these proteins relevant to this work and unobserved in the crystal structure: 1–19 for the Imp-L1 and 22–42 for the IGF-1, are depicted as dashed lines (not up to a possible real scale); IGF-1 D-domain (63–70), also not visible in the complex and not present in the des(63-70)-IGF-1 analog is omitted for figure clarity. **(B)** IGF-1:IGFBP-4 complex (PDB ID 2DSR). The IGFBP N- and C-domains, and IGF-1 are in gray, green, and magenta, respectively.

In invertebrates various insulin-like peptides (ILP) were discovered, which production and secretion depend on food availability and developmental stage, and, in contrast to humans, they signal through only one insulin receptor-like receptor (5). The bioavailability of ILPs is most probably influenced also by ILP binding proteins (IBP). Although proteins related to the IGFBP-rPs were also identified in *Crustacea* and reported to bind an ILP responsible for sexual differentiation (6), proteins corresponding to the “true” vertebrate IGFBP 1–6 were not identified in invertebrates. Instead, it seems that the function of IGFBPs is fulfilled by IBPs which are composed of two immunoglobulin-like (Ig) domains and bind ILPs (including human insulin and IGF-1) with nanomolar affinities (7). Recently, we solved the apo and holo crystal structures of 242 amino acid *Drosophila* imaginal morphogenesis protein-late 2 protein (Imp-L2) which is one of the insect IBPs (7). We have shown that the ligand (*Drosophila* ILP5 and human IGF-1) binding mode of Imp-L2 differs from that of IGFBPs. The hormone accommodates its B-helix across Imp-L2 inter-domain β-sheet, facilitating also a new arrangement of this IBP (Figure 1A) In contrast, human IGFBPs bind IGF-1 in the cleft between IGFBP *N*-terminal and *C*-terminal domains (Figure 1B) (2).

There have been recently very significant breakthroughs in structural description of insulin/IGFs:receptors interactions and their changes upon signal transduction (8, 9). However, despite all advances in structural biology, the insulin/insulin-like signaling axis contains plethora of very mobile, or disordered, protein interfaces that appear disordered and escape characterization. For example the end of the B-domain, the whole C-domain, and D-domain in IGF-1 were/are untraceable in the complexes of IGF-1 with its binding partners (7, 8).

Structural mass spectrometry (MS) approaches find applications in determination of protein-protein contacts and for structural analysis (10). There is an increasing use of chemical crosslinking in structural MS approaches that use bifunctional reagents to covalently connect interacting partners, or protein chains, located in a distance defined by the arm of the chemical linker (11). One of them: sulfo-succinimidyl 4,4′-azidopentanoate (Sulfo-SDA) is a heterobifunctional cross-linker that contains an amine-reactive *N*-hydroxysuccinimide ester and a photoactivable diazirine ring. The photoactivation of the Sulfo-SDA leads to a reactive carbene intermediate that reacts with any amino acid side chain or peptide backbone within the spacer arm distance (3.9 Å). We selected heterobifunctional SDA as it allows a sequential reaction–thus eliminating unwanted intramolecular crosslinking, and due to its non-selective, versatile characters; it is expected to react at the site of interaction regardless the availability of specific reactive group. However, regardless the progress in MS methods, identification of crosslinked species is still a challenging task. The crosslinked peptides have higher charges and are overall less abundant in the background of linear peptides. Also, a low specificities of photo-inducible cross-linkers complicate the MS analysis even further (12).

Our previous crystallographic studies on the holo Imp-L2 revealed the importance of Imp-L2 *C*-terminal (~168–242) region for specific binding of the hormones, including IGF-1. We also observed IGF-1 N-terminus in a new, never previously described in this hormone, α-helical (so-called R) conformation. However, some of the Imp-L2 side chains that could form tighter interactions within the IBP region and contribute to the stabilization of this conformation, were disordered. Therefore, we explored the MS approach with the use of Sulfo-SDA toward identification of crosslinked peptides derived from IGF-1:Imp-L2 complex interface, in order to investigate the in-solution proximity of these, possibly, structurally important side-chains, and to verify our previous observation.

## Materials and Methods

### Production of Recombinant Proteins

Human IGF-1 was purchased from Tercica Inc. Human IGF-1 lacking the C-terminal D domain amino acids PLKPAKSA (des(63-70)-IGF-1) (IGF-1 UniprotKB entry P05019, amino acids 49–110) was cloned similarly to our previous work (13). Briefly, modified pRSFDuet-1 harboring Gly-1-IGF-1 was amplified by PCR using ACYCDuetUP1 (5′-GGATCTCGACGCTCTCCCT−3′) and IGF-1del PLKPAKSA Rev (5′-GCAGGTGAATTCATTACGCGCAATACATTTCCAG−3′) primers and cloned back into NcoI/EcoRI sites of pRSFDuet-1. Expression from cloned construct produces des(63-70)-IGF-1 as a fusion with an *N*-terminal His_6_ tag, GB1 protein and tobacco etch virus (TEV) protease cleavage site (Glu-Asn-Leu-Tyr-Phe-Gln↓Gly). Des(63-70)-IGF-1was created with an additional *N*-terminal Gly-1 to facilitate TEV cleavage. Des(63-70)-IGF-1 was produced in *E. coli* BL21(DE3), purified and characterized as previously published (14).

Recombinant *Drosophila* neural/ectodermal development factor Imp-L2 was produced as described previously (7). Shortly, Imp-L2 cDNA was sub-cloned to PVL1392 plasmid (BD Biosciences). Recombinant FlashBAC virus was prepared after transfection of SF21 cells (Invitrogen). High Five cells (Invitrogen) were infected with the virus for a large-scale production of the protein. Imp-L2 was purified using Strep-Tactin column and the Strep-tag II was removed by HRV14 3C protease.

### Modification of Ligands by Sulfo-SDA and Photocrosslinking

Human IGF-1 or des(63-70)-IGF-1were modified with sulfo-succinimidyl 4,4′-azidopentanoate (Sulfo-SDA, ThermoFisher). Proteins (0.2 mM, about 100 μg) in PBS were incubated with 2 mM Sulfo-SDA for 2 h on ice. The reaction was stopped by addition of a quenching Tris/HCl buffer (pH 8) to a final 40 mM concentration. The mixture was incubated 5 min at room temperature. Modified proteins in PBS (final concentration about 0.1 mM) were separated from unreacted crosslinker using Zeba Spin Desalting Column (ThermoFisher).

Ligands (about 100 μg, 10–20 nmol) modified with Sulfo-SDA on their Lys and *N*-terminal amines were incubated with recombinant Imp-L2 (76 μg, about 3 nmol) overnight at 4°C. Proteins were photocrosslinked using irradiation at 365 nm for 5 min in a distance 1 cm from the light source (UVP Black-Ray B-100AP Lamp, Fisher Scientific).

Samples after photocrosslinking were analyzed using SDS-PAGE (14% gel). The extent of crosslinking of IGF-1 and des(63-70)-IGF-1 to Imp-L2 was determined using western-blotting with anti-IGF-1 antibody (1C5-1A2) (MA1-088, ThermoFisher). Mixture of proteins after photocrosslinking was separated on SDS-PAGE and stained with Coomassie R250. Bands containing the unmodified proteins and bands containing the crosslinked product were excised.

### MALDI-TOF/TOF

Small samples of ligands (about 10 μg, 1–2 nmol) modified with SDA were irradiated at 365 nm for 5 min in a distance 1 cm from the light source (UVP Black-Ray B-100AP Lamp, Fisher Scientific). Extent of modification of available amino groups with diazirine arm was controlled using UltrafleXtreme™ MALDI-TOF/TOF (Bruker Daltonics). SDA-modified IGF-1 (0.025 mM) was also incubated with high concentration of free amino acid (0.1 M glycine), with a tetra peptide (10 mM GFFMetF-amide) or with a peptide TFEDYLHNVVTVPRPS (0.1 mM) and irradiated.

Samples were prepared by dried droplet method. Both, unmodified and SDA-modified ligands were diluted in 50% ACN and 0.1% TFA (10 pmol/μL). Saturated DHB matrix solution (50% ACN, 0.1% TFA) was prepared. The matrix solution was mixed in equal volumes with the sample solution. The mixture was pipetted on the target (1 μL) and dried at ambient temperature.

IGF-1 and des(63-70)-IGF-1 were measured in linear mode, instrumental setting tuned to 2–20 kDa. The accelerating voltage was set at 25 kV. Typically, spectra were obtained by accumulating 5,000 shots.

### MS/MS

Bands corresponding to crosslinked protein were excised from the SDS-PAGE gels and distained. Cysteines were reduced with 50 mM DTT for 45 min at 60°C and free cysteines were alkylated with 100 mM iodoacetamide for 30 min at room temperature in the dark. Trypsin digestion proceeded overnight at 37°C with an enzyme/protein ratio of 1:20 (w/w). Peptides extracted from the gel were loaded on a trap column (ZORBAX 300SB-C18, 5 μm, 5 × 0.3 mm, Agilent, Santa Clara, CA) and desalted for 5 min at flow rate 20 μL/min. Peptides were then separated by reversed phase C18 column (ZORBAX SB C18 RR 3.5 μ 150 × 0.3 mm, Agilent, Santa Clara, CA) at a flow rate 10 μL/min using capillary HPLC system (Agilent Technologies) under the following gradient conditions: 1–10% B in 1 min, 10–45% B in 19 min, 45–95% B in 5 min, where solvent A was 0.1% formic acid, 2.0% acetonitrile in water and solvent B was 0.1% formic acid in 98% acetonitrile. The column was heated at 50°C and connected directly to an 15T solariX FT-ICR mass spectrometer (Bruker Daltonics) using an electrospray ion source. The instrument was on line calibrated resulting in mass accuracy below 2 ppm. Data acquisition and data processing were performed by ftmsControl 2.1.0 and DataAnalysis 4.2 (Bruker Daltonics).

### Strategy for Identification of Crosslinked Peptides

MS spectra were searched for peptides corresponding to the sequence of Imp-L2 and IGF-1 or des(63-70)-IGF-1 using Links software (15). The Links algorithm was set to consider the carbamidomethylation of cysteine and the possible single oxidation of methionine. The mass error threshold was kept below 2 ppm and assigned fragments were verified manually. Crosslinked peptides were searched between the two sequences as N-terminus or Lysine connected anywhere by an arm with molecular mass +82.0419 Da (16). Suggested crosslinks found in several (*n* ≥ 4) successive scans were considered as reliable and the spectra were manually searched for theoretical MS/MS fragments of peptides originating from the two proteins.

Further, the data were exported to mgf format and loaded into the StavroX software (version 3.6.0.1). The cross-linked peptides were searched using following parameters: fixed caramidomethylation of cysteines, variable oxidation of methionines, specificity of SDA for site 1 “K,S,T,Y,” specificity for site 2 “A,I,L,M,S,T,W,H,D,E,N,K,P,G,V,Q,m,C,B” (where “m” represents oxidized methionine and “B” represents caramidomethylated cysteine). Precursor precision was set to 2.0 ppm and fragment ion precision was set to 20.0 ppm. FDR cut off was below 5%. Decoy analysis was performed by shuffling fasta database while keeping the amino acids of protease sites in place (17).

## Results

### Photocrosslinking of IGF-1 or des(63-70)-IGF-1 to Imp-L2

**Figure 2:**
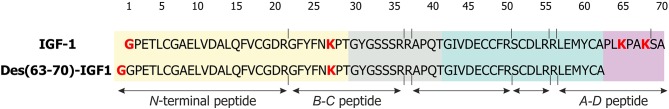
Amino-acid sequences of IGF-1 **(top)** and des(63-70)-IGF-1 **(bottom)**. IGF-1 domains are highlighted by background colors (B domain yellow, A domain blue, C domain gray and D domain violet). Trypsin cleavage sites and expected peptides are shown. Peptides containing amino acid residues with primary amines (red) susceptible to succinimide reaction are named *N*-terminal*, B-C* and *A-D*.

We prepared the IGF-1 lacking the D domain residues PLKPAKSA (des(63-70)-IGF-1) with the aim to reduce the number of primary amines available for reaction with the succinimide ester in Sulfo-SDA and thus simplify the MS analysis (Figure 2). Also, an additional *N*-terminal glycine residue Gly(-1) was incorporated into IGF-1 to facilitate production of des(63-70)-IGF-1 (13).

**Figure 3:**
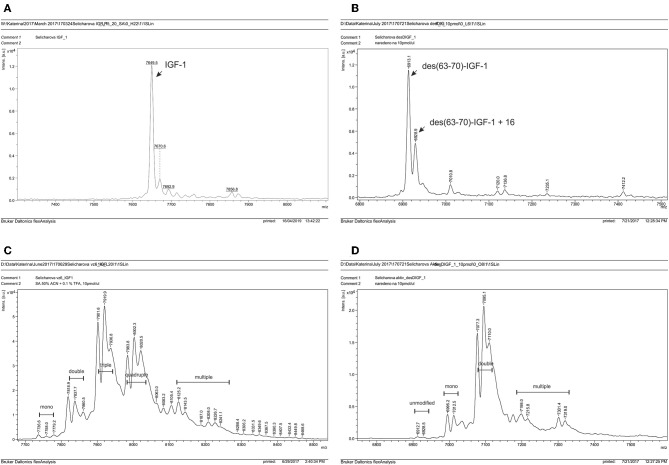
MALDI-TOF/TOF analysis of **(A)** IGF-1 and **(B)** des(63-70)-IGF-1 after coupling with Sulfo-SDA. Carbene was formed after irradiation of diazirine moiety and was either eliminated by formation of double bound resulting in the increase of Mr of about 82 Da or it reacted with one molecule of water resulting in the increase of Mr of about 100 Da. **(C)** SDA modified IGF-1. **(D)** SDA modified des(63-70)-IGF-1.

The extent of modification of the wt and truncated IGF-1 by SDA was tested using MALDI-TOF/TOF (Figure 3). The SDA-modified IGF-1 yielded triple modified product and, in slightly lower amount, quadruple modified IGF-1 as well (Figure 3C). As expected, the prevailing products of the des(63-70)-IGF-1 modification were only double modified species with two diazirine arms (Figure 3D). Species with higher extend of modification that are observed in the chromatograms (Figures 3C,D) resulted from limited reactivity of *N*-hydroxysuccinimide ester with hydroxyl group in side chains of serines, threonines, and tyrosines.

To check specificity of the crosslinking reaction, we performed control experiments where SDA-modified IGF-1 was incubated with excess of free amino acid/test peptides and irradiated. MALDI-TOF/TOF analysis was used to search for the products. Either alkene or alcohol were formed (same as in Figure 3C) after irradiation of diazirine, and no visible crosslinked products were detected in these experiments despite high concentration of peptides.

**Figure 4:**
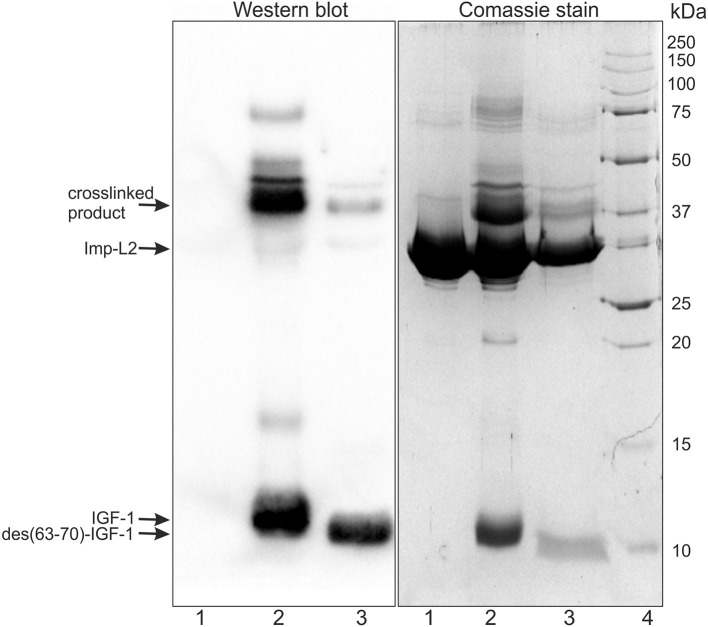
SDS-PAGE and western blot analysis of photocrosslinked IGF-1 or des(63-70)-IGF-1 to Imp-L2. Imp-L2 (line 1) was photocrosslinked to SDA-modified IGF-1 (line 2) or des(63-70)-IGF-1 (line 3). Samples were resolved by SDS-PAGE on 14% gels and stained either by Coomassie blue or blotted to PVDF membrane and detected with anti-IGF-1 antibody (1C5-1A2) (MA1-088, ThermoFisher). Molecular weight standards (BioRad) are shown in line 4.

Efficiency of Imp-L2 crosslinking reactions with IGF-1 and des(63-70)-IGF-1 were analyzed using SDS-PAGE and western-blot. Unreacted hormones and Imp-L2, together with a crosslinked product, were clearly visible on the Coomassie blue stained gels. The crosslinked product was detected with an IGF-1 antibody (Figure 4). Only about 10–20% of the starting amount of Imp-L2 was crosslinked to the hormones in both, IGF-1 and des(63-70)-IGF-1 reactions.

### Identification of Crosslinked Peptides

**Figure 5:**
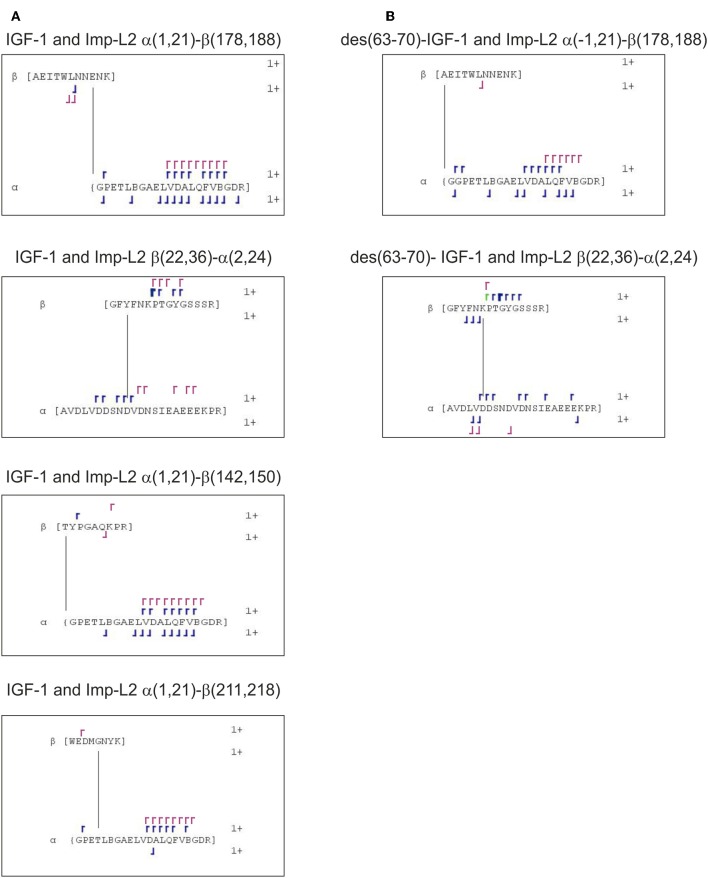
Diagrams of identified crosslinked peptides **(A)** between IGF-1:Imp-L2 and **(B)** between des(63-70)-IGF-1:Imp-L2. MS/MS fragments and sites of crosslinks found in StavroX search are shown in blue. Manually assigned fragments confirming crosslinks suggested by Links algorithm are shown in violet.

Crosslinked peptides were searched using Links algorithm (dealing with MS data) and StavroX software (dealing with MS/MS data). Both approaches identified the same crosslinks. The results are shown in Supplementary Material (“StavroX results for crosslinks between IGF-1 or des (63-70)-IGF-1 and Imp-L2.pdf” and “Links results for crosslinks between IGF-1 or des(63-70)-IGF-1 and Imp-L2.xlsx”). Crosslinked peptides are listed in Table 1 for des(63-70)-IGF-1:Imp-L2 complex and in Table 2 for IGF-1:Imp-L2 complex and diagrams showing fragments observed in respective MS/MS spectra of each peptide are drawn in Figure 5.

**Table 1:** Crosslinked peptides between des(63-70)-IGF-1 and Imp-L2.

**Crosslinked peptide (des(63-70)-IGF-1)–(Imp-L2)**	**Exp. mass**	**Thr. mass**	**Error**	**StavroX score**
(−1, 21)–(178, 188)	3776.7892	3776.7894	−0.06	67
(22, 36)–(2, 24)	4307.9865	4307.9851	+0.31	103

**Table 2:** Crosslinked peptides between IGF-1 and Imp-L2.

**Crosslinked peptide (IGF-1)–(Imp-L2)**	**Exp. mass**	**Thr. mass**	**Error**	**StavroX score**
(1, 21)–(178, 188)	3,719.7681	3719.7680	−0.04	79
(1, 21)–(211, 218)	3430.5381	3430.5388	−0.2	24
(1, 21)–(142, 150)	3405.6567	3405.6566	+0.03	61
(22, 36)–(2, 24)	4307.9859	4307.9851	+0.19	41

As expected, the crosslinking of des(63-70)-IGF-1 provided simpler data and was considered as decisive when assessing the Links data of IGF-1 analysis (see Supplementary Material). Only the crosslinks found in multiple successive scans and confirmed by manual searches for MS/MS fragments were considered as reliable. The other hits were excluded as false positive. All the selected identified crosslinks were subsequently also found through automated StavroX analysis.

We have not identified any reliable crosslinked product of Lys residues within the IGF-1 D domain. Although there were multiple suggested crosslinks in the Links search none fulfilled the criteria of reliability, and no ions confirming the crosslink were found. No potential crosslinks in D domain were identified using the StavroX. The D domain probably did not contribute to the ligand interaction.

**Figure 6:**
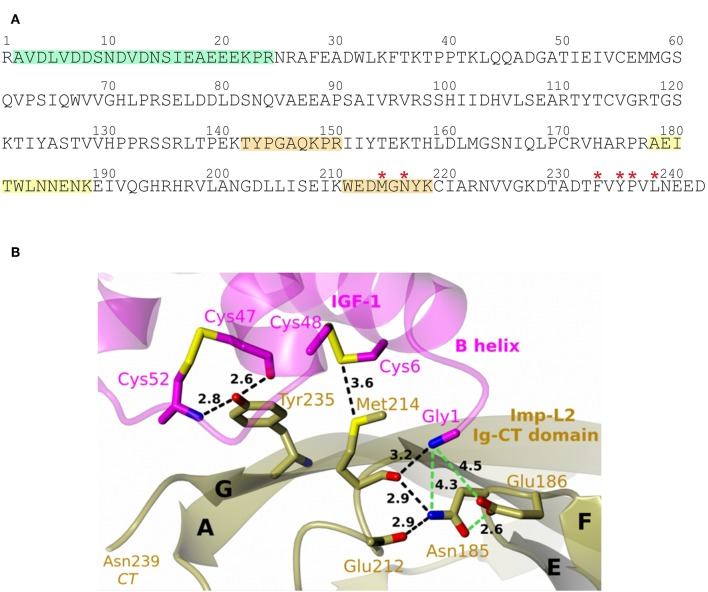
**(A)** Amino acid sequence of Imp-L2. Peptides crosslinked to IGF-1and des(63-70)-IGF1 are highlighted by background colors (IGF-1 peptide (22–36) green and *N*-terminal peptide yellow for both ligands and orange for IGF-1). Key residues for hormone binding observed in holo-Imp-L2 structure are indicated by red stars (7). **(B)** Some of the des(63-70)-IGF-1:Imp-L2 contacts identified in this work and in the crystal structure (PDB ID 6FF3) of this complex. IGF-1 and Imp-L2 are in magenta and green, respectively; sulfur atoms in yellow, oxygen in red, nitrogens in blue; hydrogen bonds and close Van der Waals contacts as black dashed lines, and putative inter-atom contacts relevant for this work are in green dashed lines.

The des(63-70)-IGF-1 *N*-terminal Gly(-1) was found to be bound to Imp-L2 peptide 178–188 (AEITWLNNENK). Identical crosslinked peptide was found for IGF-1:Imp-L2 experiment (Figure 6). We observed the b6 ion of peptide 178–188 Imp-L2 which would suggest that the crosslink was formed within the sequence 184–188 of Imp-L2 (Figure 5 and Supplementary Material). This finding corresponds to the crystallography data as IGF-1 Gly1 is very close to Imp-L2 Asn185 (~4.3 Å) and Glu186 (~4.5 Å) (Figure 6B).

Besides, other contacts of IGF-1 Gly1 were found (Table 2 and Figure 6B). The *N*-terminal peptide was crosslinked to the peptide 211–218 (WEDMGNYK) which was identified as major binding interaction site in the X-ray structure. It was also crosslinked to peptide 142–150 (TYPGAQKPR). All three Imp-L2 peptides found to be crosslinked to IGF-1 N-terminus form neighboring β-strands A, G, and F (Figures 1A, 6B) of Imp-L2 Ig *C*-terminal domain.

Next, we identified crosslinked peptide connecting IGF-1 23–36 with Imp-L2 peptide 2–24 (AVDLVDDSNDVDNSIEAEEEKPR) (Tables 1, 2, Figure 5 and Supplementary Material). The crosslinked peptide was identified in both IGF-1:Imp-L2 and des(63-70)-IGF-1:Imp-L2 crosslinking experiments. The 22–44 region of IGF-1 and the *N*-terminal part of Imp-L2 (1-29) were not visible in the crystal structure despite their spatial proximity (Figure 1A) (7). However, a high number of scans (see Supplementary Material) in which this crosslinked peptide was identified suggests its relative abundance and, consequently, would indicate strong interactions within these sequences. As shown in our experiments with test peptides, the carbene formed upon photoactivation of diazirine would react readily with water to create alcohol, or rearrange to give an alkene if there is no close contact with an interacting partner thus reducing the yield of the crosslinking reaction (16).

## Discussion

Structural mass spectrometry (MS) approaches find utilization in determination of protein-protein contacts and for structural analysis. We aimed to explore the cross-linking/mass spectrometry approach toward revealing the in-solution proximity of protein-protein interface. We utilized a IGF-1:Imp-L2 complex that we previously studied using X-ray crystallography. The knowledge of the structure allowed us to critically evaluate reliability of crosslinked peptides proposed by the scoring algorithm of MS spectra but also to verify our previous observation. To simplify the MS analysis we prepared the IGF-1 lacking the D domain. As anticipated, the MS spectrum of des(63-70)-IGF-1 crosslinked to Imp-L2 was simpler, clearer and contained less false-positive hits compared to the IGF-1:Imp-L2 spectrum. Nevertheless, identical crosslinked peptides were identified for both wt and truncated IGF-1 bound to Imp-L2 which further supported reliability of the approach.

We previously described a unique R-state like rearrangement of N-terminus of IGF-1 firmly anchored to Imp-L2 β-sheet surface in the IGF-1:Imp-L2 crystal structure (7). This arrangement was supported by the crosslinking experiments (Figure 6) where we identified the *N*-terminal peptide of IGF-1 crosslinked to the *C*-terminal part of Imp-L2.

Surprisingly the most distinctive crosslinked peptide was found within the *N*-terminal peptide 2–24 of Imp-L2 and 23–36 region of IGF-1. These parts of the proteins were not observed in the crystal structure. This would suggest that they may contribute to IBP:hormone complex formation but such interaction is transient due to mobility of this region. We have previously shown that the increase of the separation of Imp-L2 N-(1-30) and C-termini (236–238) upon hormone binding allows the expansion of the β-sheet surface in its holo crystal structure. We also postulated that the 1–16 polypeptide of the Imp-L2 (unobserved in the crystal structure) is involved in triggering of the apo → holo transition upon “pressure” from the incoming hormone. This subsequently involves increased contacts between N-termini and 70–90 loop of the Imp-L2, and formation of newly arranged Imp-L2 dimer with broad hormone-binding surfaces (7). Here, our crosslinking MS data confirm the predicted “hormone sensing” role of 1–16 *N*-terminal part of the Imp-L2, which occurs in concert with similar, but more directional and contact specific, hormone:Imp-L2 C-terminus interactions. However, much lower sequence similarity of the *N*-terminal (~1–30) part of insects IBPs (7) in comparison with rather conserved C-termini of these proteins suggest different roles of these hormone:IBP contacts. For example, hormone:IBP N-terminus interactions may be responsible for the initiation of the formation of this complex and priming IBP for an effective engagement with the ligand (“entropic” contribution), while hormone:IBP C-terminus contacts assure firmness/stability of this complex (“enthalpic” contribution).

Photo-crosslinking has already been used in mapping the interactions of ligands (insulin/IGF) with their receptors and binding proteins (18, 19). The incorporation of *para*-azido-Phe photo-probe into hormones was employed. In addition, the hormones had to be specifically labeled, mostly biotinylated, to enable detection of the site of crosslink after partial proteolysis. Although these studies provided substantial input toward our understanding of hormone:receptor binding, these experiments were rather complex, and allowed mainly only an approximate estimation of the site of crosslink (19). Our work presented here shows that the MS based techniques may surpass, to great extent, such obstacles although they still present some—but different–limitations. It seems that the main bottleneck here is the detection and identification of the crosslinked peptides. The applications of various scoring procedures with statistical methods in automated data analysis does not prevent misassignment of the crosslinked products (12). To address this issue we utilized our knowledge of the mass of the crosslinker arm and the span/length of the expected peptides originating from the ligand. These were exploited in the input for the search of reliable outputs, allowing us to eliminate some false-positive hits. Obtained crosslinked peptides were confirmed by MS/MS data. However, even though crosslinked proteins were separated on SDS-PAGE, the abundance of crosslinked peptides in the spectra was low. Consequently the suggested sites of crosslink given by StavroX (Figure 5) should be considered with caution as MS/MS sequence coverage of the peptides was insufficient in most of the cases.

To summarize, we have shown nice convergence of crystallographic and crosslinking data in solving the interaction between IGF-1 and Imp-L2, and in clarification of postulated, but not observed in solid state, hormone:IBP interfaces. Hence this MS-based approach and methodology can be utilized in other systems where contributing peptides from one interacting partner can be clearly defined and the crosslinked product could be isolated.

## Data Availability Statement

The datasets generated for this study are available on request to the corresponding author.

## Author Contributions

PP performed the MS and analyzed the data. CV prepared the recombinant Imp-L2. JR and JL prepared the recombinant des(63-70)-IGF-1. JJ designed the experiment and edited the manuscript. AB correlated the crosslinking data with crystallography data and wrote the manuscript. IS designed and performed the experiments, evaluated and interpreted the data, and wrote the manuscript.

### Conflict of Interest

The authors declare that the research was conducted in the absence of any commercial or financial relationships that could be construed as a potential conflict of interest.
