# Supplementary material for: Cross-Linking/Mass Spectrometry Uncovers Details of Insulin-Like Growth Factor Interaction With Insect Insulin Binding Protein Imp-L2
Source: Front Endocrinol (Lausanne). 2019 Oct 9;10:695. doi: 10.3389/fendo.2019.00695 (PMC6794382; doi:10.3389/fendo.2019.00695)
Supplement: Supplementary file 2 [file Data_Sheet_1.PDF]

Crosslinked peptide between IGF-1 and Imp-L2
(1,21)-(142,150)

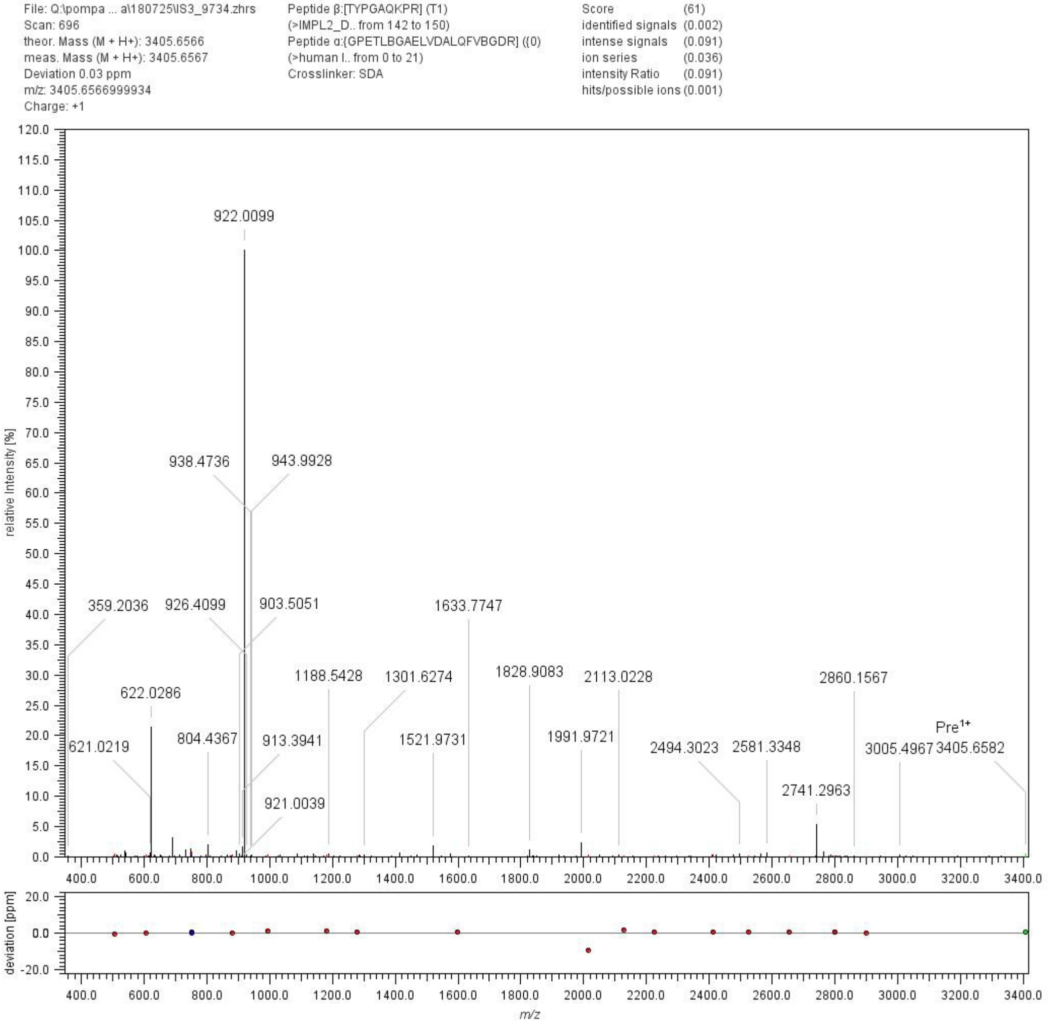

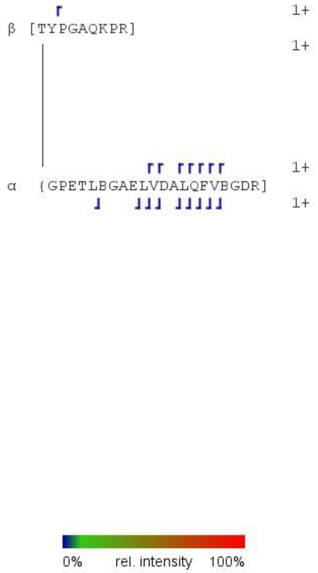

b	b-H2O	b-NH3	AA	y	y-H2O	y-NH3
Peptide: α						
Charge: +1						
1156.6108	1138.6004	1139.5844	G			
1253.6637	1235.6531	1236.6371	P	2250.0529	2232.0424	2233.0264
1382.7063	1364.6957	1365.6797	E	2153.0002	2134.9896	2135.9735
1483.7539	1465.7434	1466.7274	T	2023.9576	2005.947	2006.931
1596.838	1578.8275	1579.8115	L	1922.9099	1904.8993	1905.8833
1756.8687	1738.8581	1739.8421	B	1809.8258	1791.8153	1792.7993
1813.8901	1795.8796	1796.8636	G	1649.7952	1631.7846	1632.7686
1884.9273	1866.9167	1867.9007	A	1592.7737	1574.7632	1575.7472
2013.9699	1995.9593	1996.9433	E	1521.7366	1503.726	1504.7101
2127.0539	2109.0433	2110.0274	L	1392.694	1374.6833	1375.6675
2226.1223	2208.1118	2209.0958	V	1279.6099	1261.5994	1262.5834
2341.1493	2323.1387	2324.1227	D	1180.5415	1162.531	1163.515
2412.1864	2394.1758	2395.1598	A	1065.5146	1047.5039	1048.488
2525.2704	2507.2599	2508.2439	L	994.4775	976.4669	977.4509
2653.329	2635.3185	2636.3024	Q	881.3934	863.3828	864.3669
2800.3974	2782.3869	2783.3709	F	753.3348	735.3243	736.3083
2899.4659	2881.4553	2882.4393	V	606.2664	588.2559	589.2399
3059.4965	3041.4859	3042.4699	B	507.198	489.1874	490.1715
3116.518	3098.5074	3099.4914	G	347.1674	329.1567	330.1408
3231.5449	3213.5342	3214.5184	D	290.1459	272.1352	273.1193
			R	175.119	157.1084	158.0924
Peptide: β						
Charge: +1						
2490.1639	2472.1534	2473.1374	T			
2653.2273	2635.2167	2636.2007	Y	916.4999	898.4894	899.4734
2750.28	2732.2695	2733.2534	P	753.4366	735.426	736.41
2807.3015	2789.2909	2790.2749	G	656.3837	638.3733	639.3573
2878.3386	2860.328	2861.3121	A	599.3624	581.3518	582.3357
3006.3972	2988.3866	2989.3706	Q	528.3253	510.3147	511.2987
3134.4921	3116.4816	3117.4656	K	400.2667	382.2561	383.2401
3231.5449	3213.5342	3214.5184	P	272.1717	254.1612	255.1452
			R	175.119	157.1084	158.0924

Crosslinked peptide between IGF-1 and Imp-L2
(1,21)-(211,218)

File: Q:\pompa ... a\180725\IS3_9734.zhrs
Scan: 764
theor. Mass (M + H+): 3430.5388
meas. Mass (M + H+): 3430.5381
Deviation -0.2 ppm
m/z: 3430.53814428835
Charge: +1

Peptide β:[WEDMGNYK] (G5)
(>IMPL2_D.. from 211 to 218)
Peptide α:{GPETLBGAELVDALQFVBGDR] (T4)
(>human I.. from 0 to 21)
Crosslinker: SDA

Score	(24)
identified signals	(0.04)
intense signals	(0.091)
ion series	(0.035)
intensity Ratio	(0.091)
hits/possible ions	(0.011)

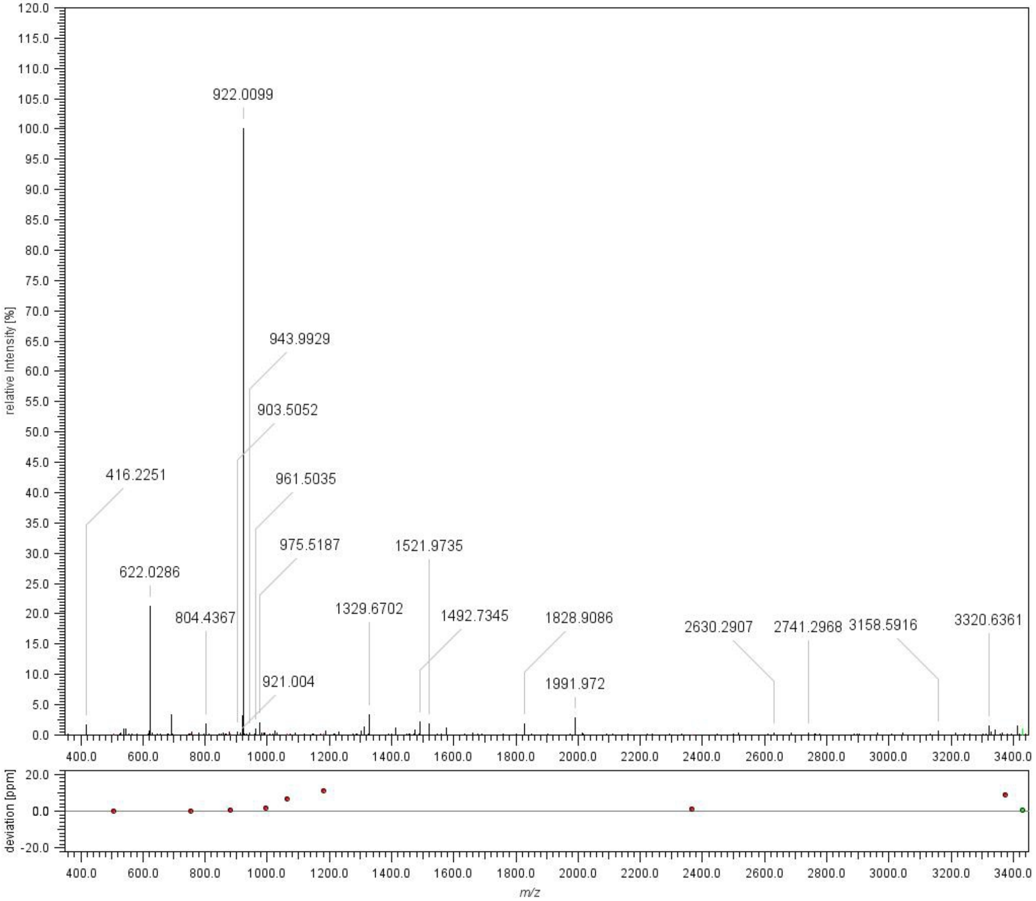

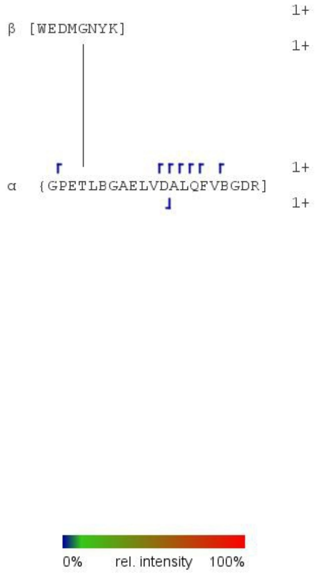

b	b-H2O	b-NH3	AA	y	y-H2O	y-NH3
Peptide: α						
Charge: +1						
58.0287	40.0182	41.0022	G			
155.0815	137.0709	138.055	P	3373.5174	3355.5068	3356.4908
284.1241	266.1135	267.0975	E	3276.4645	3258.454	3259.4381
1508.6362	1490.6257	1491.6097	T	3147.422	3129.4115	3130.3955
1621.7203	1603.7097	1604.6937	L	1922.9099	1904.8993	1905.8833
1781.7509	1763.7404	1764.7244	B	1809.8258	1791.8153	1792.7993
1838.7724	1820.7618	1821.7458	G	1649.7952	1631.7846	1632.7686
1909.8095	1891.7989	1892.783	A	1592.7737	1574.7632	1575.7472
2038.8521	2020.8415	2021.8256	E	1521.7366	1503.726	1504.7101
2151.9362	2133.9256	2134.9096	L	1392.694	1374.6833	1375.6675
2251.0046	2232.994	2233.978	V	1279.6099	1261.5994	1262.5834
2366.0315	2348.021	2349.005	D	1180.5415	1162.531	1163.515
2437.0686	2419.0581	2420.0421	A	1065.5146	1047.5039	1048.488
2550.1527	2532.1421	2533.1262	L	994.4775	976.4669	977.4509
2678.2113	2660.2007	2661.1846	Q	881.3934	863.3828	864.3669
2825.2797	2807.2691	2808.2531	F	753.3348	735.3243	736.3083
2924.3481	2906.3375	2907.3216	V	606.2664	588.2559	589.2399
3084.3788	3066.3682	3067.3521	B	507.198	489.1874	490.1715
3141.4002	3123.3897	3124.3737	G	347.1674	329.1567	330.1408
3256.4272	3238.4166	3239.4006	D	290.1459	272.1352	273.1193
			R	175.119	157.1084	158.0924
Peptide: β						
Charge: +1						
187.0866	169.076	170.06	W			
316.1292	298.1186	299.1026	E	3244.4595	3226.449	3227.433
431.1561	413.1456	414.1296	D	3115.4169	3097.4064	3098.3904
562.1966	544.186	545.1701	M	3000.39	2982.3793	2983.3634
3007.3271	2989.3165	2990.3005	G	2869.3494	2851.3389	2852.323
3121.37	3103.3594	3104.3434	N	424.2191	406.2085	407.1925
3284.4333	3266.4227	3267.4068	Y	310.1761	292.1656	293.1496
			K	147.1128	129.1022	130.0863

Crosslinked peptide between IGF-1 and Imp-L2
(1,21)-(178,188)

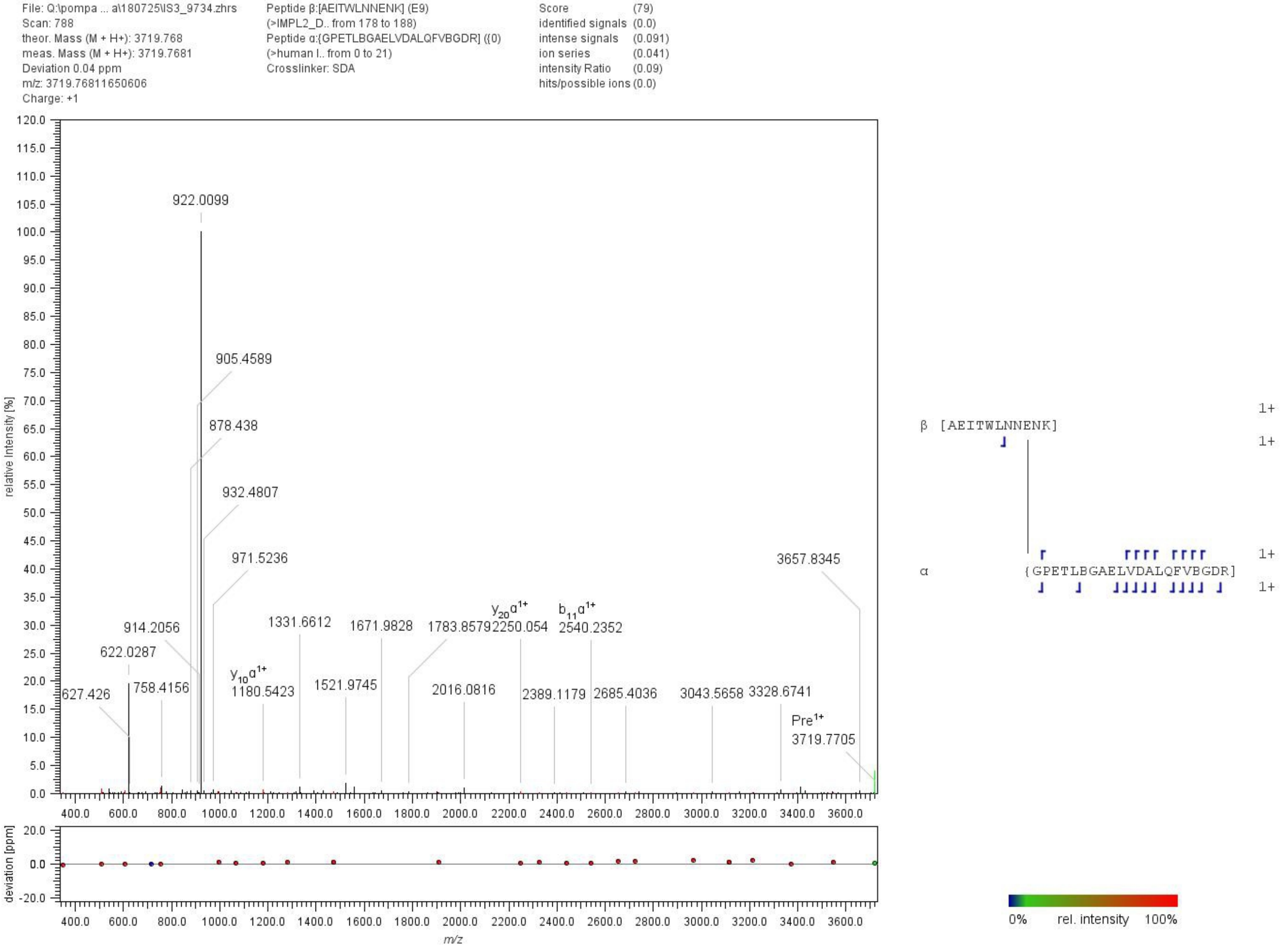

b	b-H2O	b-NH3	AA	y	y-H2O	y-NH3
Peptide: α						
Charge: +1						
1470.7222	1452.7118	1453.6958	G			
1567.7751	1549.7645	1550.7485	P	2250.0529	2232.0424	2233.0264
1696.8177	1678.8071	1679.7911	E	2153.0002	2134.9896	2135.9735
1797.8654	1779.8548	1780.8388	T	2023.9576	2005.947	2006.931
1910.9494	1892.9389	1893.9229	L	1922.9099	1904.8993	1905.8833
2070.9801	2052.9695	2053.9535	B	1809.8258	1791.8153	1792.7993
2128.0015	2109.991	2110.975	G	1649.7952	1631.7846	1632.7686
2199.0387	2181.0281	2182.0121	A	1592.7737	1574.7632	1575.7472
2328.0813	2310.0707	2311.0547	E	1521.7366	1503.726	1504.7101
2441.1653	2423.1547	2424.1388	L	1392.694	1374.6833	1375.6675
2540.2337	2522.2232	2523.2072	V	1279.6099	1261.5994	1262.5834
2655.2607	2637.2501	2638.2341	D	1180.5415	1162.531	1163.515
2726.2978	2708.2872	2709.2712	A	1065.5146	1047.5039	1048.488
2839.3818	2821.3712	2822.3553	L	994.4775	976.4669	977.4509
2967.4404	2949.4299	2950.4139	Q	881.3934	863.3828	864.3669
3114.5088	3096.4983	3097.4823	F	753.3348	735.3243	736.3083
3213.5773	3195.5667	3196.5506	V	606.2664	588.2559	589.2399
3373.6079	3355.5973	3356.5814	B	507.198	489.1874	490.1715
3430.6294	3412.6188	3413.6028	G	347.1674	329.1567	330.1408
3545.6563	3527.6457	3528.6298	D	290.1459	272.1352	273.1193
			R	175.119	157.1084	158.0924
Peptide: β						
Charge: +1						
72.0444	54.0338	55.0178	A			
201.087	183.0764	184.0603	E	3648.7309	3630.7203	3631.7043
314.171	296.1605	297.1445	I	3519.6883	3501.6777	3502.6617
415.2187	397.2081	398.1922	T	3406.6042	3388.5936	3389.5777
601.298	583.2875	584.2714	W	3305.5565	3287.546	3288.53
714.3821	696.3715	697.3556	L	3119.4771	3101.4667	3102.4507
828.425	810.4145	811.3985	N	3006.3932	2988.3826	2989.3666
942.468	924.4574	925.4414	N	2892.3502	2874.3397	2875.3237
3459.6195	3441.609	3442.593	E	2778.3073	2760.2967	2761.2808
3573.6625	3555.6519	3556.6359	N	261.1557	243.1452	244.1292
			K	147.1128	129.1022	130.0863

Crosslinked peptide between IGF-1 and Imp-L2
(22,36)-(2,24)

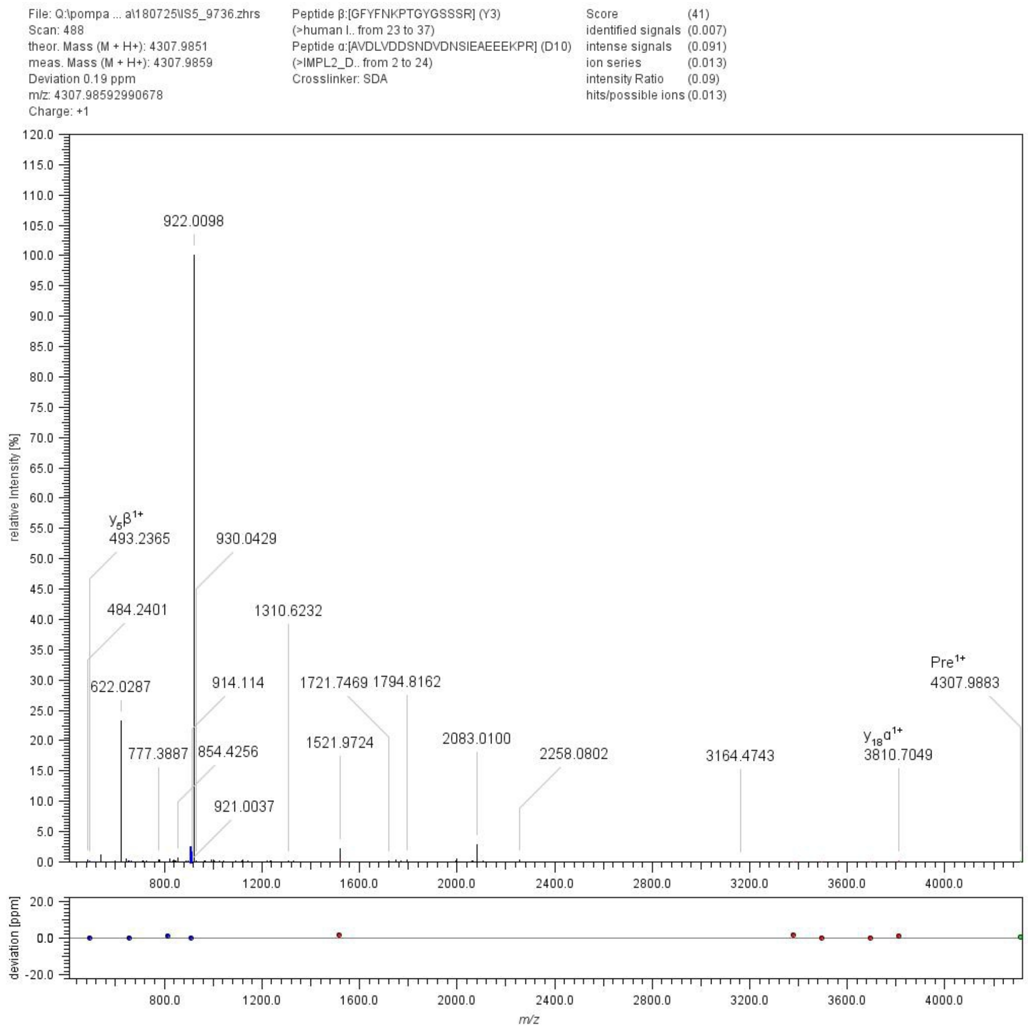

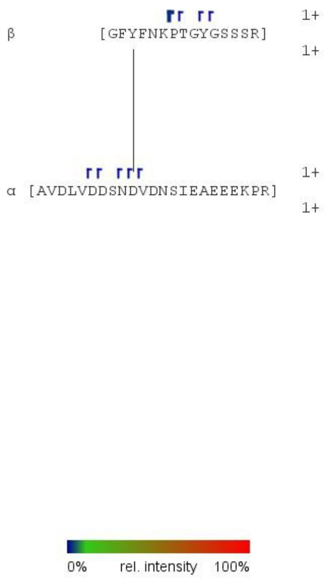

b	b-H2O	b-NH3	AA	y	y-H2O	y-NH3
Peptide: α						
Charge: +1						
72.0444	54.0338	55.0178	A			
171.1128	153.1022	154.0863	V	4236.948	4218.9374	4219.9214
286.1397	268.1292	269.1132	D	4137.8796	4119.8690	4120.853
399.2238	381.2131	382.1973	L	4022.8526	4004.8421	4005.8261
498.2922	480.2817	481.2657	V	3909.7686	3891.758	3892.742
613.3192	595.3086	596.2926	D	3810.7002	3792.6896	3793.6736
728.3461	710.3355	711.3196	D	3695.6732	3677.6627	3678.6467
815.3781	797.3676	798.3516	S	3580.6463	3562.6357	3563.6197
929.4211	911.4105	912.3945	N	3493.6142	3475.6037	3476.5877
2793.2638	2775.2533	2776.2372	D	3379.5713	3361.5608	3362.5448
2892.3323	2874.3217	2875.3057	V	1515.7285	1497.718	1498.702
3007.3592	2989.3485	2990.3327	D	1416.6601	1398.6496	1399.6335
3121.402	3103.3916	3104.3756	N	1301.6332	1283.6226	1284.6066
3208.4342	3190.4236	3191.4076	S	1187.5903	1169.5797	1170.5637
3321.5182	3303.5077	3304.4917	I	1100.5582	1082.5477	1083.5317
3450.5608	3432.5502	3433.5343	E	987.4742	969.4636	970.4476
3521.5979	3503.5874	3504.5714	A	858.4316	840.421	841.405
3650.6405	3632.63	3633.614	E	787.3945	769.3839	770.3679
3779.6831	3761.6725	3762.6566	E	658.3519	640.3413	641.3253
3908.7257	3890.7151	3891.6992	E	529.3093	511.2987	512.2827
4036.8207	4018.8101	4019.7941	K	400.2667	382.2561	383.2401
4133.8734	4115.8629	4116.8468	P	272.1717	254.1612	255.1452
			R	175.119	157.1084	158.0924
Peptide: β						
Charge: +1						
58.0287	40.0182	41.0022	G			
205.0971	187.0866	188.0706	F	4250.9636	4232.9531	4233.9371
3008.3643	2990.3538	2991.3377	Y	4103.8952	4085.8847	4086.8687
3155.4328	3137.4222	3138.4062	F	1300.628	1282.6175	1283.6015
3269.4757	3251.4651	3252.4491	N	1153.5596	1135.5491	1136.5331
3397.5706	3379.5601	3380.5441	K	1039.5166	1021.5061	1022.4901
3494.6234	3476.6128	3477.5969	P	911.4217	893.4112	894.3952
3595.6711	3577.6605	3578.6445	T	814.369	796.3584	797.3424
3652.6926	3634.682	3635.666	G	713.3212	695.3107	696.2947
3815.7559	3797.7453	3798.7293	Y	656.2998	638.2893	639.2732
3872.7773	3854.7668	3855.7508	G	493.2365	475.2259	476.21
3959.8094	3941.7988	3942.7828	S	436.215	418.2045	419.1884
4046.8414	4028.8308	4029.8149	S	349.183	331.1724	332.1565
4133.8734	4115.8629	4116.8468	S	262.151	244.1404	245.1244
			R	175.119	157.1084	158.0924

Crosslinked peptide between des(63-70)-IGF-1 and Imp-L2 (-1,21)-(178,188)

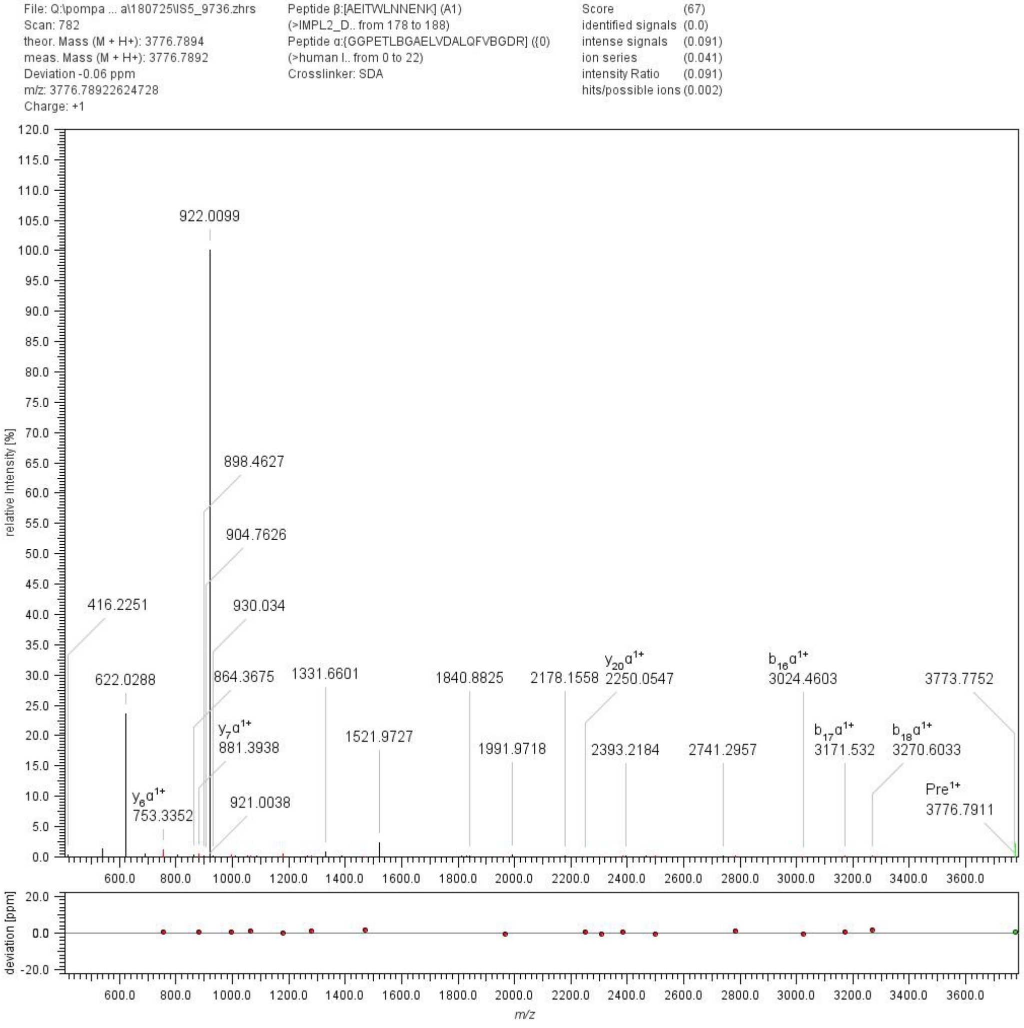

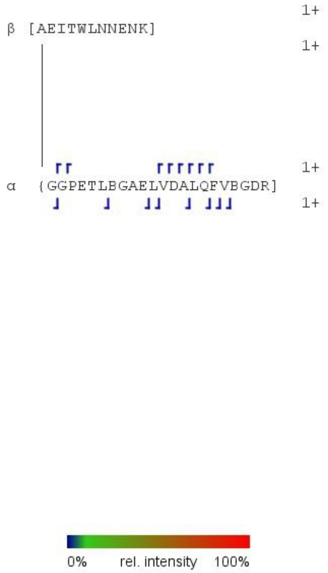

b	b-H2O	b-NH3	AA	y	y-H2O	y-NH3
Peptide: α						
Charge: +1						
1470.7222	1452.7118	1453.6958	G			
1527.7438	1509.7331	1510.7172	G	2307.0744	2289.0638	2290.0478
1624.7965	1606.786	1607.77	P	2250.0529	2232.0424	2233.0264
1753.8392	1735.8286	1736.8126	E	2153.0002	2134.9896	2135.9735
1854.8868	1836.8763	1837.8603	T	2023.9576	2005.947	2006.931
1967.9709	1949.9603	1950.9443	L	1922.9099	1904.8993	1905.8833
2128.0015	2109.991	2110.975	B	1809.8258	1791.8153	1792.7993
2185.023	2167.0124	2167.9965	G	1649.7952	1631.7846	1632.7686
2256.0601	2238.0496	2239.0336	A	1592.7737	1574.7632	1575.7472
2385.1027	2367.0922	2368.0762	E	1521.7366	1503.726	1504.7101
2498.1868	2480.1762	2481.1601	L	1392.694	1374.6833	1375.6675
2597.2552	2579.2446	2580.2286	V	1279.6099	1261.5994	1262.5834
2712.2821	2694.2716	2695.2556	D	1180.5415	1162.531	1163.515
2783.3192	2765.3087	2766.2927	A	1065.5146	1047.5039	1048.488
2896.4033	2878.3927	2879.3768	L	994.4775	976.4669	977.4509
3024.4618	3006.4513	3007.4353	Q	881.3934	863.3828	864.3669
3171.5303	3153.5197	3154.5038	F	753.3348	735.3243	736.3083
3270.5987	3252.5882	3253.5722	V	606.2664	588.2559	589.2399
3430.6294	3412.6188	3413.6028	B	507.198	489.1874	490.1715
3487.6508	3469.6403	3470.6243	G	347.1674	329.1567	330.1408
3602.6778	3584.6672	3585.6512	D	290.1459	272.1352	273.1193
			R	175.119	157.1084	158.0924
Peptide: β						
Charge: +1						
2517.1747	2499.1643	2500.1483	A			
2646.2174	2628.2069	2629.1909	E	1260.6219	1242.6113	1243.5953
2759.3015	2741.2909	2742.2749	I	1131.5793	1113.5687	1114.5527
2860.3492	2842.3386	2843.3226	T	1018.4952	1000.4847	1001.4687
3046.4285	3028.4179	3029.4019	W	917.4476	899.437	900.421
3159.5125	3141.502	3142.486	L	731.3682	713.3577	714.3416
3273.5555	3255.5449	3256.5288	N	618.2842	600.2736	601.2576
3387.5984	3369.5878	3370.5719	N	504.2413	486.2307	487.2147
3516.641	3498.6304	3499.6144	E	390.1983	372.1878	373.1718
3630.6839	3612.6734	3613.6574	N	261.1557	243.1452	244.1292
			K	147.1128	129.1022	130.0863

Crosslinked peptide between des(63-70)-IGF-1 and Imp-L2 (22,36)-(2,24)

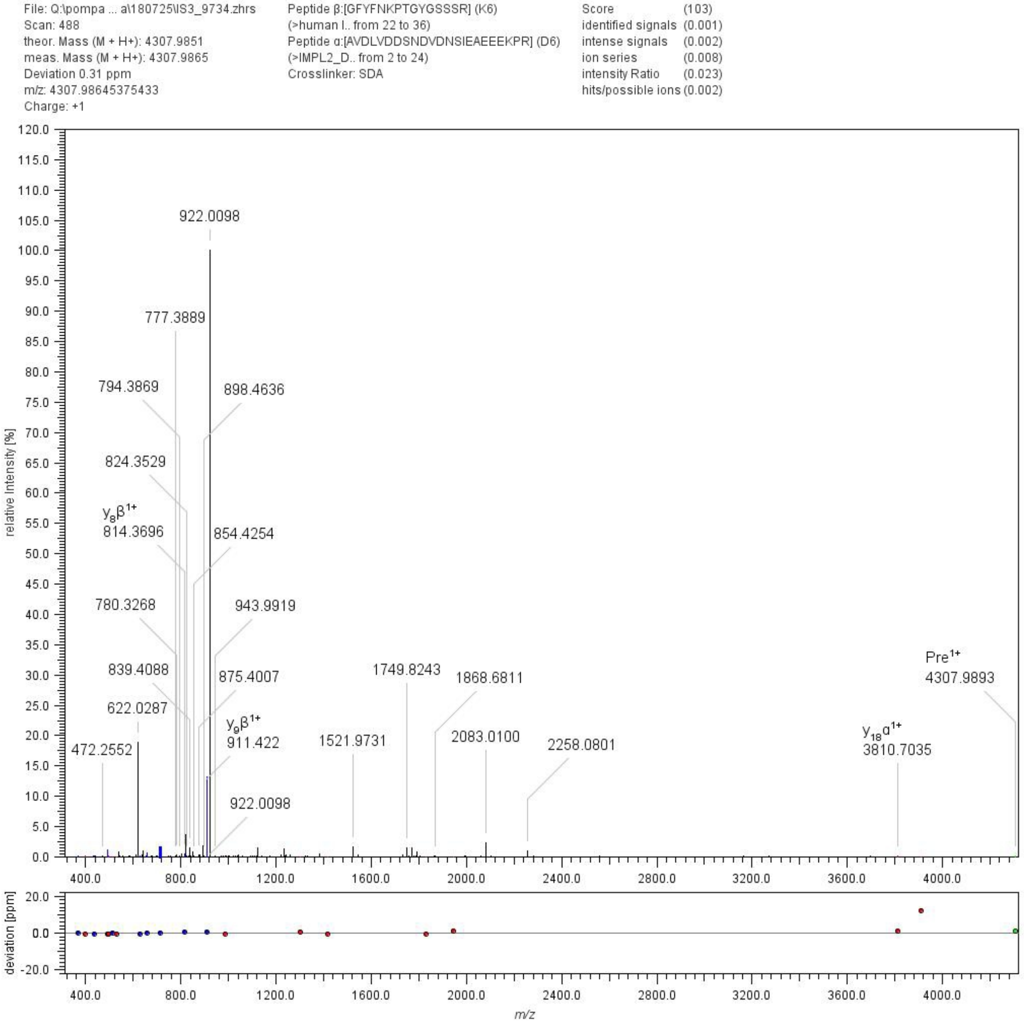

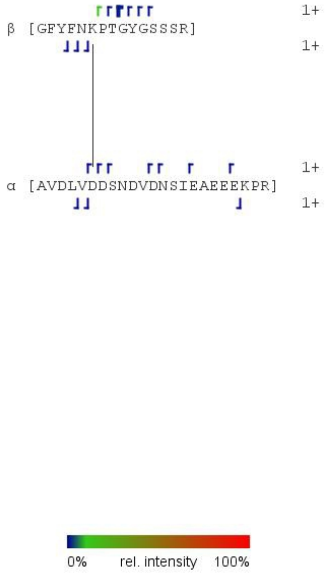

b	b-H2O	b-NH3	AA	y	y-H2O	y-NH3
Peptide: α						
Charge: +1						
72.0444	54.0338	55.0178	A			
171.1128	153.1022	154.0863	V	4236.948	4218.9374	4219.9214
286.1397	268.1292	269.1132	D	4137.8796	4119.8690	4120.853
399.2238	381.2131	382.1973	L	4022.8526	4004.8421	4005.8261
498.2922	480.2817	481.2657	V	3909.7686	3891.758	3892.742
2362.135	2344.1244	2345.1085	D	3810.7002	3792.6896	3793.6736
2477.1619	2459.1514	2460.1354	D	1946.8574	1928.8468	1929.8308
2564.194	2546.1834	2547.1674	S	1831.8304	1813.8199	1814.8039
2678.2369	2660.2262	2661.2104	N	1744.7984	1726.7878	1727.7719
2793.2638	2775.2533	2776.2372	D	1630.7555	1612.7449	1613.7289
2892.3323	2874.3217	2875.3057	V	1515.7285	1497.718	1498.702
3007.3592	2989.3485	2990.3327	D	1416.6601	1398.6496	1399.6335
3121.402	3103.3916	3104.3756	N	1301.6332	1283.6226	1284.6066
3208.4342	3190.4236	3191.4076	S	1187.5903	1169.5797	1170.5637
3321.5182	3303.5077	3304.4917	I	1100.5582	1082.5477	1083.5317
3450.5608	3432.5502	3433.5343	E	987.4742	969.4636	970.4476
3521.5979	3503.5874	3504.5714	A	858.4316	840.421	841.405
3650.6405	3632.63	3633.614	E	787.3945	769.3839	770.3679
3779.6831	3761.6725	3762.6566	E	658.3519	640.3413	641.3253
3908.7257	3890.7151	3891.6992	E	529.3093	511.2987	512.2827
4036.8207	4018.8101	4019.7941	K	400.2667	382.2561	383.2401
4133.8734	4115.8629	4116.8468	P	272.1717	254.1612	255.1452
			R	175.119	157.1084	158.0924
Peptide: β						
Charge: +1						
58.0287	40.0182	41.0022	G			
205.0971	187.0866	188.0706	F	4250.9636	4232.9531	4233.9371
368.1605	350.1499	351.1339	Y	4103.8952	4085.8847	4086.8687
515.2288	497.2183	498.2023	F	3940.8319	3922.8213	3923.8054
629.2718	611.2613	612.2453	N	3793.7635	3775.7529	3776.7369
3397.5706	3379.5601	3380.5441	K	3679.7206	3661.71	3662.694
3494.6234	3476.6128	3477.5969	P	911.4217	893.4112	894.3952
3595.6711	3577.6605	3578.6445	T	814.369	796.3584	797.3424
3652.6926	3634.682	3635.666	G	713.3212	695.3107	696.2947
3815.7559	3797.7453	3798.7293	Y	656.2998	638.2893	639.2732
3872.7773	3854.7668	3855.7508	G	493.2365	475.2259	476.21
3959.8094	3941.7988	3942.7828	S	436.215	418.2045	419.1884
4046.8414	4028.8308	4029.8149	S	349.183	331.1724	332.1565
4133.8734	4115.8629	4116.8468	S	262.151	244.1404	245.1244
			R	175.119	157.1084	158.0924
